# Supplementary material for: Partitioning gene-based variance of complex traits by gene score regression
Source: PLoS One. 2020 Aug 20;15(8):e0237657. doi: 10.1371/journal.pone.0237657 (PMC7446906; doi:10.1371/journal.pone.0237657)
Supplement: S1 File — (PDF) [file pone.0237657.s001.pdf]

Partitioning gene-based variance of complex traits by gene score regression

Figures

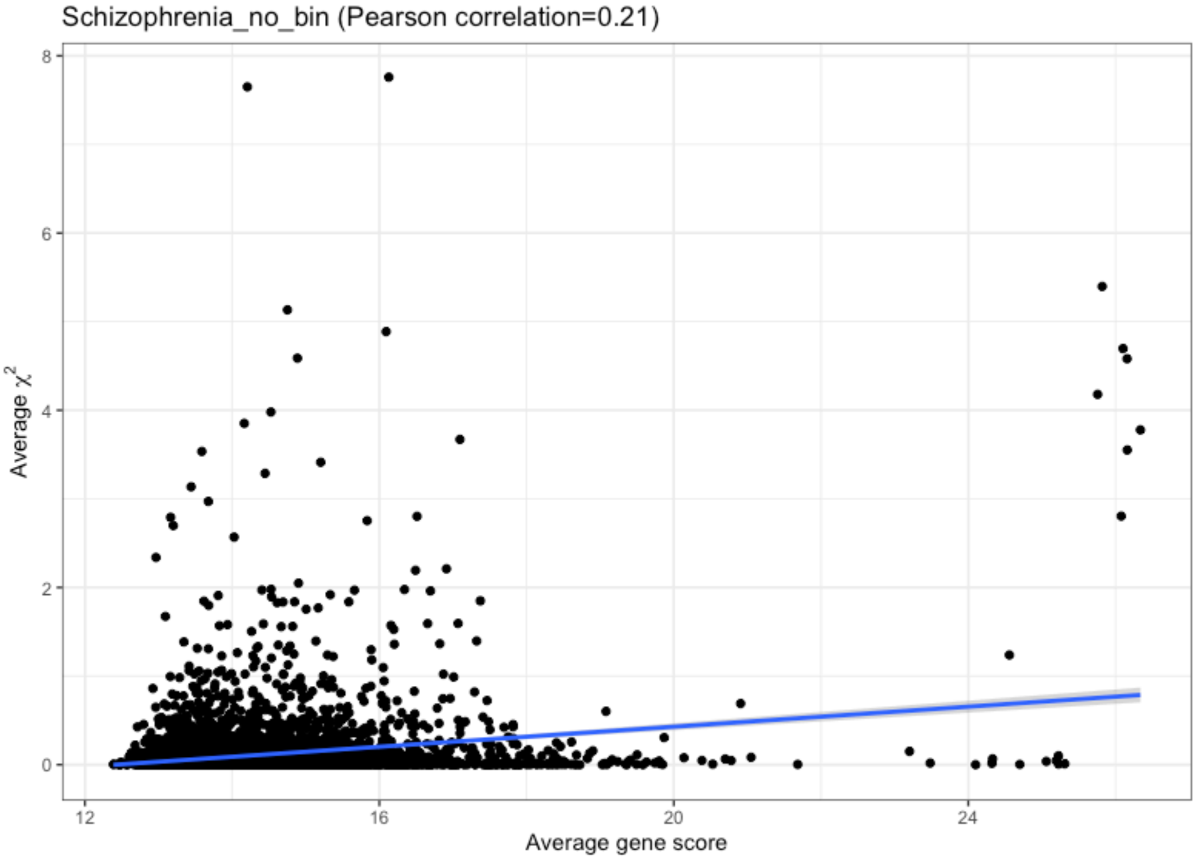

S1 Fig: Gene scores were correlated with marginal TWAS chi-square statistics of Schizophrenia. An binned version is supplied in Fig 2. The fitted slope is 0.044 (SD = 0.0039) compared to 0.056 (SD = 0.0035), fitted by binned gene scores and χ^2.

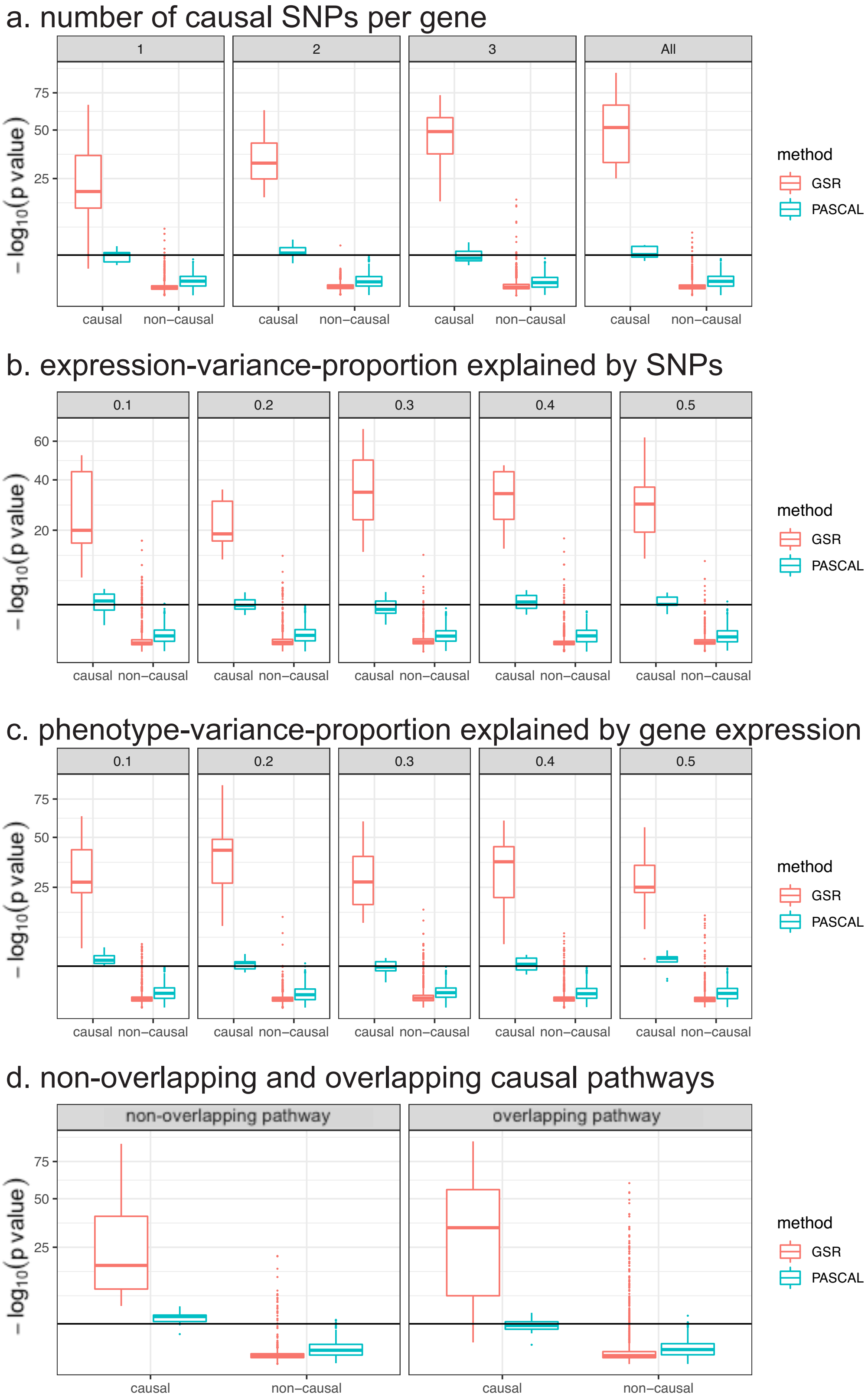

S2 Fig: Simulation to evaluate methods in various simulation settings. We compared GSR and PASCAL [1] working with (a) different number of causal SNP per gene (b) different SNP-gene heritability; (3) different gene-phenotype variance explained; and (d) different overlapping causal pathway. For visibility of the large p-values, we plot the y-axis on the scale of the squared root of $-log_{10}$(p-values).

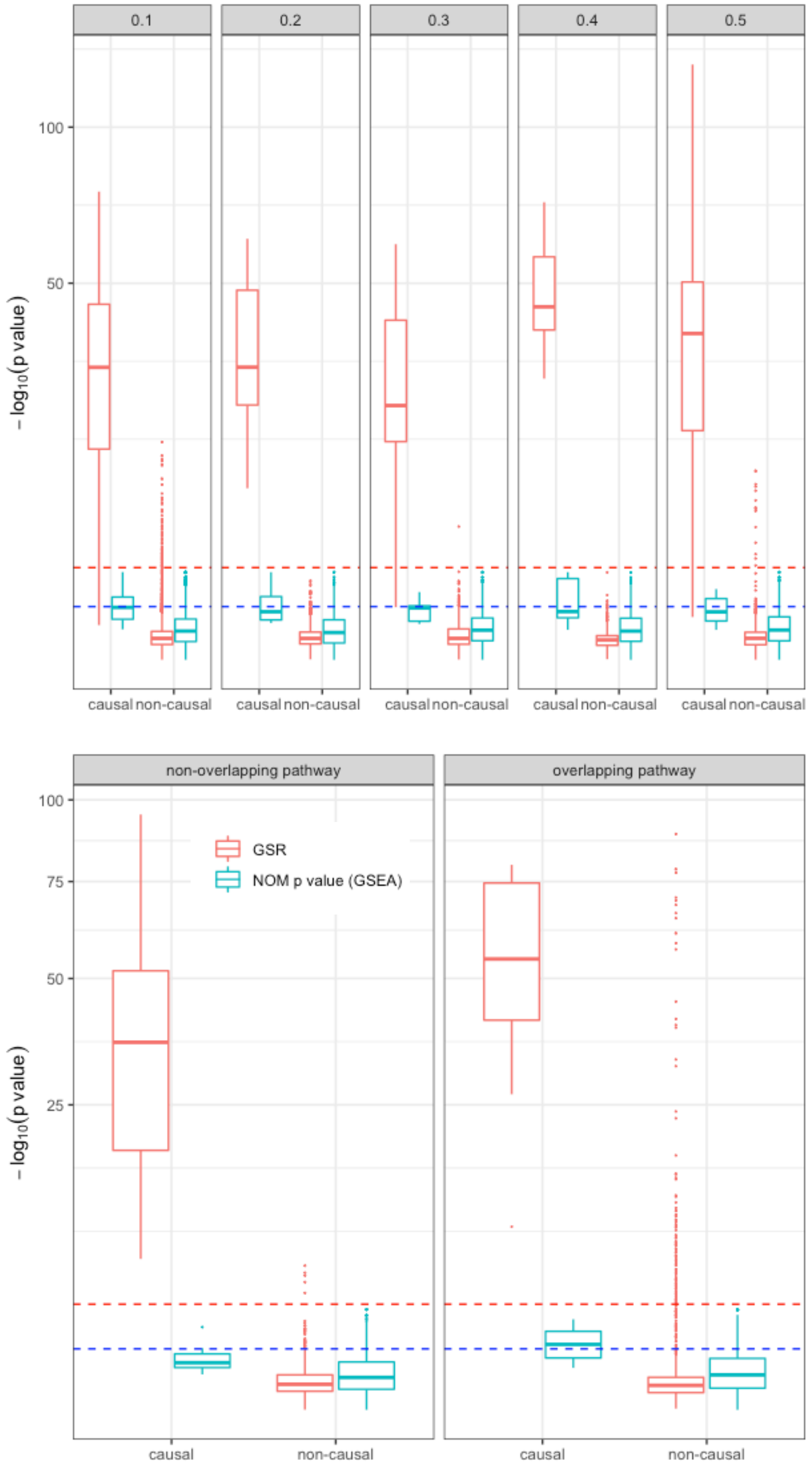

S3 Fig: Comparison of pathway enrichment between GSR and GSEA [2]. Pathway enrichment was inferred using observed gene expression.

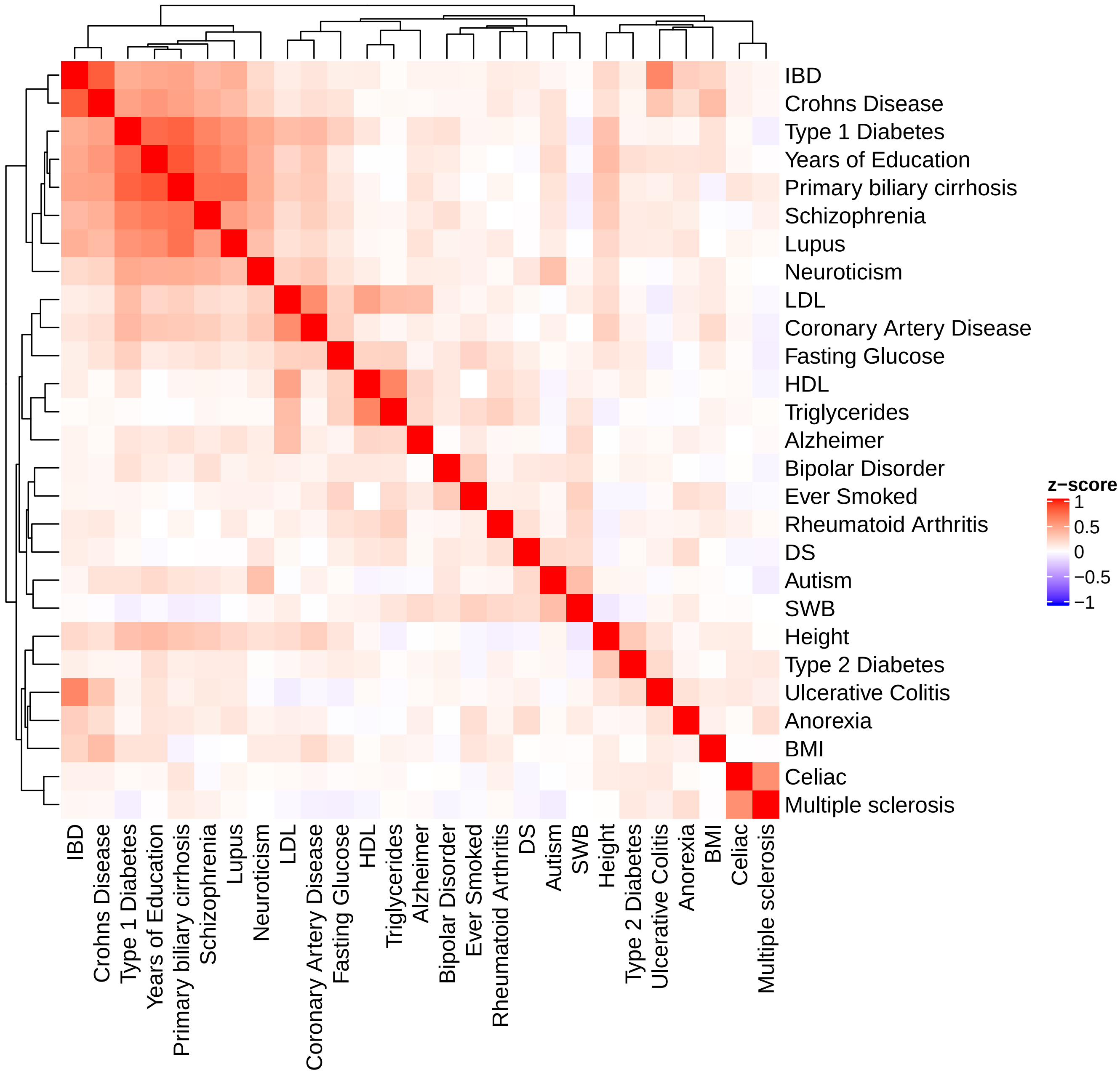

S4 Fig: Gene set enrichments suggest shared genetic and biological pathways by related complex traits

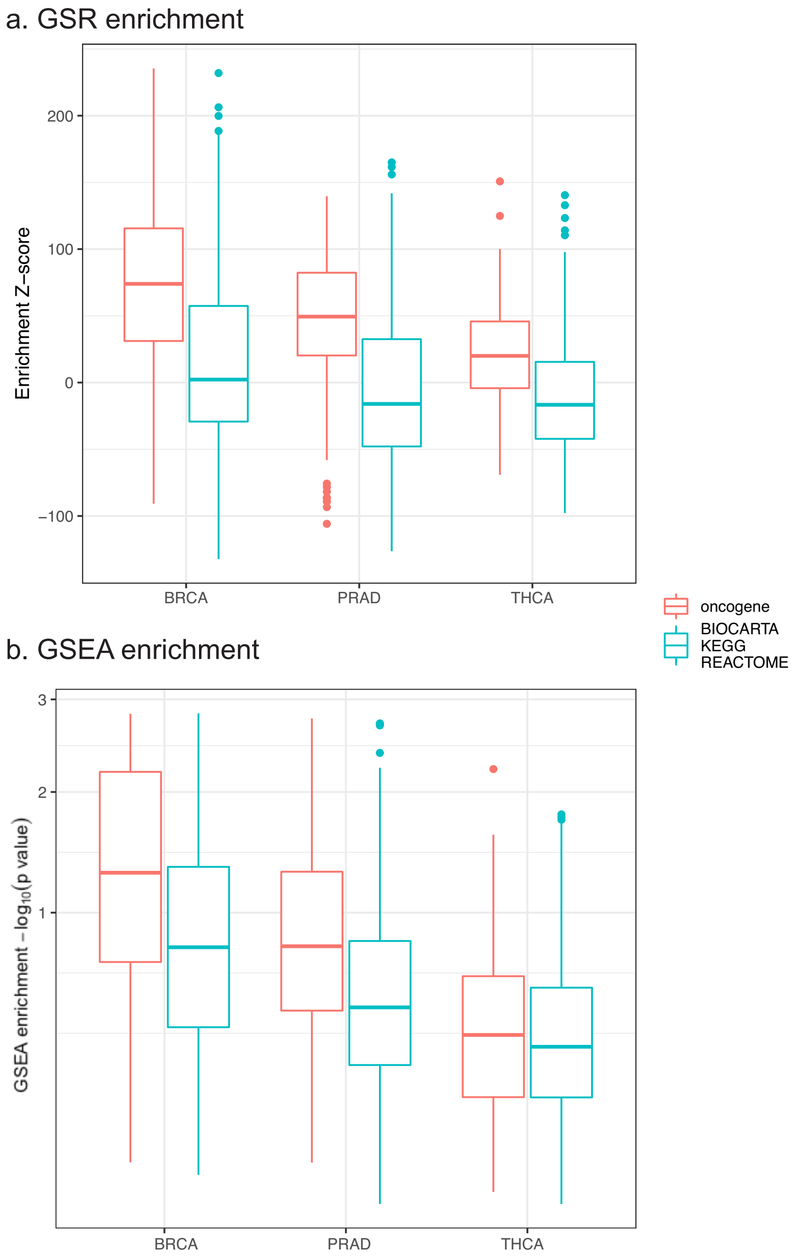

S5 Fig: Tumour-specific gene set enrichment. GSR and GSEA were performed separately on the processed gene expression data of BRCA, PRAD and THCA samples versus normal samples from TCGA and GTEx. We tested the enrichments for oncogenic signatures as well as 1,050 gene sets obtained from BIOCARTA, KEGG and REACTOME. We then separately plotted the enrichment Z-scores for oncogenic gene sets and the general gene sets across the three tumour types.

Table

S1 Table: Gene set enrichments on select traits. The enrichments were applied to three gene sets: (1) 4,360 GO Biology Processes terms from MSigDb; (2) combined 1,051 BIOCARTA, KEGG, REACTOME gene sets from MSigDb; and (3) 207 gene sets each derived from GTEx and Franke tissue/cell-specifically expressed genes.

trait	gene set	p-value	t-value
HDL	REACTOME_CHYLOMICRON_MEDIATED_LIPID_TRANSPORT	3.23e-151	26.98
HDL	GO_NEUTRAL_LIPID_CATABOLIC_PROCESS	1.70e-132	25.14
HDL	GO_TRIGLYCERIDE_CATABOLIC_PROCESS	1.70e-132	25.14
HDL	GO_MACROMOLECULAR_COMPLEX_REMODELING	5.82e-131	24.98
HDL	GO_ACYLGLYCEROL_HOMEOSTASIS	1.11e-113	23.17
HDL	REACTOME_LIPOPROTEIN_METABOLISM	3.99e-112	23.00
HDL	REACTOME_LIPID_DIGESTION_MOBILIZATION_AND_TRANSPOR	5.93e-96	21.17
HDL	GO_PROTEIN_LIPID_COMPLEX_SUBUNIT_ORGANIZATION	2.58e-90	20.51
HDL	REACTOME_PACKAGING_OF_TELOMERE_ENDS	2.65e-42	13.74
HDL	KEGG_PPAR_SIGNALING_PATHWAY	1.72e-32	11.94
Lupus	GO_POSITIVE_REGULATION_OF_INTERFERON_ALPHA_PRODUCT	7.06e-150	26.85
Lupus	GO_POSITIVE_REGULATION_OF_INTERFERON_BETA_PRODUCTI	1.46e-101	21.82
Lupus	GO_RESPONSE_TO_MURAMYL_DIPEPTIDE1	2.26e-96	21.22
Lupus	GO_POSITIVE_REGULATION_OF_INTERLEUKIN_12_PRODUCTIO	1.39e-78	19.05
Lupus	GO_REGULATION_OF_INTERFERON_ALPHA_PRODUCTION	1.38e-72	18.27
Lupus	REACTOME_INTERFERON_GAMMA_SIGNALING	2.90e-65	17.28
Lupus	REACTOME_INTERFERON_ALPHA_BETA_SIGNALING	1.30e-29	11.36
Lupus	KEGG_TOLL_LIKE_RECEPTOR_SIGNALING_PATHWAY	1.70e-24	10.26
Lupus	REACTOME_INTERFERON_SIGNALING	1.18e-13	7.44
Lupus	REACTOME_CYTOKINE_SIGNALING_IN_IMMUNE_SYSTEM	5.11e-07	5.03

References

[1] David Lamparter, Daniel Marbach, Rico Rueedi, Zoltán Kutalik, and Sven Bergmann. Fast and Rigorous Computation of Gene and Pathway Scores from SNP-Based Summary Statistics. *PLoS Computational Biology*, 12(1):e1004714–20, January 2016.

[2] Aravind Subramanian, Pablo Tamayo, Vamsi K Mootha, Sayan Mukherjee, Benjamin L Ebert, Michael A Gillette, Amanda Paulovich, Scott L Pomeroy, Todd R Golub, Eric S Lander, and Jill P Mesirov. Gene set enrichment analysis: a knowledge-based approach for interpreting genome-wide expression profiles. *Proceedings of the National Academy of Sciences of the United States of America*, 102(43):15545–15550, October 2005.
