# Supplementary material for: Partitioning gene-based variance of complex traits by gene score regression
Source: PLoS One. 2020 Aug 20;15(8):e0237657. doi: 10.1371/journal.pone.0237657 (PMC7446906; doi:10.1371/journal.pone.0237657)
Supplement: Attachment — Submitted filename: Partitioning gene-based variance of complex traits by gene score regression.docx [file pone.0237657.s002.docx]

In this manuscript, the authors proposed a method, Gene Score Regression (GSR), to estimate the phenotypic variance explained by the gene expression and can be used to test for gene set or pathway enrichments based on GWAS summary statistics or the observed gene expression. They performed simulation experiments and also applied GSR to real data. The results supported that GSR is powerful and robust. My major concern is power and false positive rate. In simulation study, the authors only performed 10 times for each setting, if computation time is not a problem, it would be good to perform at least 1000 times to estimate power and false positive rate for some settings, particularly low heritability. Minor concern is the format. It seems that the authors use another format in the beginning but didn’t completely match PLOS ONE’s format. It really confused me when I reviewed this manuscript, so I think the authors should reorganize it. Figure quality and errors should also be careful. I listed my questions as following by section:

1 Introduction

1) “In TWAS, we can regress on the expression changes using the genotype information from the reference cohort…” In this description, the dependent variable is the expression changes and the independent variable is the genotype information. According to the cited reference 10, Figure 2 and Equation 1, the dependent variable is the expression, not the expression changes. Please clarify.
2) In Figure 1 (a), please specific which SNP is 1, 2 and 3, respectively? In (b), the blue SNPs are causal for a non-causal gene. Are the two blue SNPs the most significant SNPs among two non-causal genes, respectively? In (c), what does the number represent?

2. Related Methods

3) “…it does not account for the gene-gene correlation, which is distinct from TWAS-induced correlation but is rather due to the sharing of transcriptional regulatory network among genes.” Please explain again that what leads to TWAS-induced correlation? (LD or other factors)

3. Methods

3.1 Phenotypic variance explained by gene expression

4) For Eq (2), does it still hold for binary outcome (y), i.e., logistic regression?
5) Please verify Eq (4) and (5) for the term of $\mathbf{A}_{g}^{\mathrm{gwas}}\mathbf{y}$ and $\mathbf{A}_{g}\mathbf{y}$. For OLS solution, they will ${\boldsymbol{(}\mathbf{A}_{g}^{\mathrm{gwas}}\boldsymbol{)}}^{T}\mathbf{y}$.and $\mathbf{A}_{g}^{T}\mathbf{y}$, respectively.
6) The gene expression of GWAS ($\mathbf{A}_{g}^{\mathrm{gwas}}$) were estimated based on a reference panel. This estimation is reliable for “controls” in GWAS that represent generation population as those from the reference panel. Is it still reliable for “cases” in GWAS using estimated values ($\hat{W}_{g}$) from the reference panel.
7) Please define the notions for i and j.
8) The Eq (6) was not used in the following text. What is the purpose to show $\hat{\beta}$ in the Eq(6)?
9) “From (8) to (9), we assume that all of the random variables are independent. The assumption holds if gene causal effects are independent and are also independent from the gene-gene correlation.” If assumption doesn’t hold, what kind of bias will be introduced (e.g., underestimate or overestimate)?
10) Please explain how to obtain $\chi_{g}^{2}$. Does it the test statistic from the regression of phenotype ***y*** on gene expression (or predicted gene expression)?

3.2 Partitioning variance component by gene sets

11) “The full derivation is similar to that for Eq (12) and detailed in Supplementary Methods.” Please indicate which section in Supplementary Methods.
12) “Therefore, we regress one gene set at a time along with a dummy gene set that include the union of all of the genes in the gene sets. The dummy gene set is used to account for unbalanced gene sets.” In a regression model for one gene set, does it include two independent variables, i.e., one is the gene score for a given gene set and the other is the gene score for a dummy gene set? In the dummy gene set, does it include genes belonging to the gene set of interest? Why a dummy gene set can be used to account for unbalanced gene sets? Please explain.
13) According to the main equation, an intercept in a regression model would be close to 1. In what condition, the intercept will be away from 1? In the text, “We also include an intercept in the regression model to properly control non-gene-set biases.” How to control non-gene-set biases?

3.3 Gene score regression on total gene expression

14) In page 5, $\hat{\alpha}_{g}=\frac{1}{N_{gwas}}\hat{A}_{g}^{T}y$, but here $\hat{\alpha}_{g}=A_{g}^{T}y$. Is it correct without a term of $\frac{1}{N_{real}}$?
15) “If one gene is a causal gene and the other is not, we will see inflated summary statistic for the non-causal gene, thereby confounding the detection for causal pathways.”. Does it indicate that the non-causal gene will be detected and the false positive rate then increases in this case?

3.4 Simulation

Simulation step 1: simulate gene expression:

16) In 1000 Genomes Project, there are 503 individuals of European ancestry. What are the exclusion criteria to remove individuals? And, how many independent blocks were generated?
17) Genotype was standardized in the reference panel, i.e. 489 individuals, or after simulation in 500 individuals. Please clarify.
18) Was a bootstrap technique used to simulate 500 individuals from 489 Europeans in 1000 Genome data?
19) “We randomly sampled k in-cis causal SNPs per gene within ±500 kb around the gene, where k = 1 (default).” If k >1, are the randomly sampled k causal SNPs per gene independent (LD r^2^<0.2)?

3.5 Data sets and 3.6 Running existing methods

20) Different methods require different data type (such as summary statistics or individual genotype/expression data for SNP or gene level) to perform analysis. Please indicate what data type that these five methods in Table 1 require and what datasets that they used for analysis.
21) Please provide the sample size information of dataset that were used in this manuscript, including TWAS, GWAS, TCGA and GTEx.

4. Results

4.1 Gene scores correlate with TWAS statistics in polygenic complex traits

22) Please reorganize the 4.1 section. Some parts of description should be presented in the method section.
23) “We calculated TWAS marginal statistic as the product of GWAS summary statistic and eQTL weights derived from the GTEx whole blood samples.” This sentence indicates how to calculate TWAS marginal statistic for each SNP. However, GSR was proposed for gene level analysis, so please explain how to calculate TWAS marginal statistic for a given gene, e.g. summation of the products of GWAS summary statistic and eQTL weights within a given gene.
24) “This implies a pervasive confounding impacts on the downstream analysis using the TWAS statistic (Figure 1e) when using existing approaches that mostly assume independence of genes.” Please provide some examples for downstream analysis. And, Figure 1e should be Figure 2e
25) For Figure 2 (a), please explain why gene bin was used to show the correlation? Why not directly use gene score and chi squared? For (b), the correlation of gene score and TWAS marginal statistic is negative for T2D. How to interpret the negative correlation and what confounders could lead to the negative correlation? Figure panel names doesn’t match to the description in the figure legend.

4.2 GSR improves pathway enrichment power

26) In Table 1, what does “Exprs” refer?
27) In Figure 3, please indicate what blue and red dotted lines represent. For y axis, the label doesn’t match the description in the figure legend. Here, is the “enrichment score” (in the description) shown as the p-value?
28) “Notably,… enrichment test.” This whole paragraph should be moved to discussion section, since there is a section called “Discussion and Conclusion”.

4.3 Improved power in pathway enrichment when using the observed gene expression

29) “To evaluate the accuracy of this application, we simulated gene expression and phenotype for 1000 individuals, which were provided as input to GSR for pathway enrichment analysis.” Is it another simulation study other than section 4.2? If yes, please describe the procedures in the simulation section.

4.4 Gene set enrichments in complex traits

30) In this section, the description related to Materials and Methods should be reorganized.
31) In Figure 5, it is very unclear to directly put pathway names onto the main figure area, e.g. a band on the bottom misleading the number of gene set to test. On the right tail of Figure 5, i.e., significant for FOCUS, not for GSR, it shows that some gene set/pathway enrichments were detected by FOCUS, but not by GSR. Does it imply that GSR is not powerful in some cases? If yes, please try to evaluate this limitation.
32) For cell-type-specific enrichment analyses, was $\hat{W}_{g}$ estimated from gene expressions of specific cell type?

4.5 Application on observed gene expression

33) Please specify sample size for each cancer, including case and control, in the observed gene expression analyses.

Supplementary information

34) There are duplications in Supplementary information and Methods. Please reorganize.
