# Supplementary material for: Partitioning gene-based variance of complex traits by gene score regression
Source: PLoS One. 2020 Aug 20;15(8):e0237657. doi: 10.1371/journal.pone.0237657 (PMC7446906; doi:10.1371/journal.pone.0237657)
Supplement: Attachment — Submitted filename: Partitioning gene-based variance of complex traits by gene score regression 2nd.docx [file pone.0237657.s004.docx]

The authors clarified my questions and reorganized well. I just have four minor questions as follows.

**Methods**

Partitioning gene-based variance of complex traits

1. “…,we will be able to perform linear regression and derive regression coefficient that is an estimate for each τ_c_, respectively.” Please indicate what dependent and independent variables are for the linear regression.

Simulation design

2. In simulation step 1.3, for 500 individuals, did you sample each block for each individual with replacement? For example, for individual *i* (I = 1, …, 500) and block *j* (*j* = 1, …, 100), you randomly sampled one block *j* from 489 blocks of *j*. And, you concatenated sampled block 1, …, 100 for individual *i*. Please provide a clear description.

**Results**

3. For Figure 2, I think it would be good to add the explanation of “how to interpret negative correlation for T2D in the figure description.
4. In Table 1, “*Summary statistics” and “^†^For custom gene sets,…” are footnotes and should not be put in the table title.
