# Supplementary material for: Partitioning gene-based variance of complex traits by gene score regression
Source: PLoS One. 2020 Aug 20;15(8):e0237657. doi: 10.1371/journal.pone.0237657 (PMC7446906; doi:10.1371/journal.pone.0237657)
Supplement: Attachment — Submitted filename: plos_one_response_letter_rev2.docx [file pone.0237657.s005.docx]

Reviewer #2

The authors clarified my questions and reorganized well. I just have four minor questions as follows.

- Thank you for your feedback.

**Methods**

Partitioning gene-based variance of complex traits

1. “…,we will be able to perform linear regression and derive regression coefficient that is an estimate for each τ_c_, respectively.” Please indicate what dependent and independent variables are for the linear regression.

- We have indicated now in line 79. We regressed Chi-squares (dependent variable) on gene scores (independent variables).

Simulation design

2. In simulation step 1.3, for 500 individuals, did you sample each block for each individual with replacement? For example, for individual *i* (I = 1, …, 500) and block *j* (*j* = 1, …, 100), you randomly sampled one block *j* from 489 blocks of *j*. And, you concatenated sampled block 1, …, 100 for individual *i*. Please provide a clear description.

- For LD block j (j in {1,...,100}) of an individual i, we randomly sampled from the 489 available samples for block j, and concatenated these sampled LD blocks 1,...,100 for this individual. We repeated this procedure to simulate genotype **X**_ref for N_ref = 500 individuals as a reference population
- We have further clarified this in lines 118-121.

**Results**

3. For Figure 2, I think it would be good to add the explanation of “how to interpret negative correlation for T2D in the figure description.

- The negative correlation for T2D indicates that this trait, possibly due to complicated genetic architecture and confounding gene-to-environment interaction and drug effects, is not suitable for using our approach.
- We have added the rationale in the figure legend.

4. In Table 1, “*Summary statistics” and “^†^For custom gene sets,…” are footnotes and should not be put in the table title.

- Thank you. We have moved those to the footnotes.
